# Efficacy of an Integrated Hands-On Thyroid Ultrasound Session for Medical Student Education

**DOI:** 10.7759/cureus.12421

**Published:** 2021-01-01

**Authors:** Anisley Valenciaga, Ryan J Ivancic, Raheela Khawaja, David P Way, David P Bahner

**Affiliations:** 1 Internal Medicine, The Ohio State University Wexner Medical Center, Columbus, USA; 2 Otolaryngology - Head and Neck Surgery, The Ohio State University Wexner Medical Center, Columbus, USA; 3 Endocrinology, Diabetes and Metabolism, The Ohio State University Wexner Medical Center, Columbus, USA; 4 Emergency Medicine, The Ohio State University, Columbus, USA; 5 Emergency Medicine, The Ohio State University Wexner Medical Center, Columbus, USA

**Keywords:** thyroid, ultrasound, medical student training, education

## Abstract

As ultrasound has gained popularity with improving technology and ease-of-use, a push has been made to integrate ultrasound into the medical school curriculum. Many institutions are reporting one- to four-year integrated ultrasound curricula to augment anatomy and pathophysiology teaching. Our goal was to integrate a thyroid ultrasound scanning session into the endocrinology block of our institution’s medical school curriculum to enhance medical student understanding of thyroid anatomy and pathophysiology. We conducted a prospective, single-center cohort (pre-experimental) study to evaluate student performance and knowledge acquisition using a pretest-posttest design. These multimodal sessions, consisting of a didactic, hands-on scanning sessions, and knowledge integration tests, covered ultrasound technique and thyroid evaluation and advanced to diagnosing an abnormal thyroid and working up a thyroid nodule. There were 26 to 27 second-year medical students per session who rotated between three stations proctored by credentialled physicians. Students participated in hands-on scanning of patients with or without thyroid pathology at each station. Out of the 209 students who participated in the ultrasound sessions, 114 (54.5%) consented to participate in the research project and completed both the pretest and posttest. Test data from the 114 students showed a mean pretest score of 57.5% ± 14.6% and the mean posttest score of 73.9% ± 17.4%. They had a 16.5% ± 19.6% (p < 0.001) increase in score between the two tests. Our study demonstrates that a multimodal thyroid ultrasound scanning session is an effective tool to augment the medical school endocrinology curriculum and to improve students’ knowledge of thyroid anatomy, pathophysiology, and diagnostic workup of thyroid nodules.

## Introduction

Ultrasound has gained popularity as a relatively safe way to evaluate pathology and to aid in certain procedures [1]. Ultrasound machines are becoming increasingly more portable and with greater image quality. These factors have made clinical ultrasound a tool used by many and a field of continued growth [2,3]. In the past, ultrasound was performed primarily by the imaging technician in a specialized setting. Currently, ultrasound is used at bedside as a point-of-care measure by physicians and trainees, and physicians now rely on ultrasound to improve the diagnosis of multiple conditions at the bedside [1,3]. Furthermore, ultrasound has promoted patient safety in certain invasive procedures, such as central line placements and centeses for fluid drainage [2].

Since bedside ultrasound has become such a commonly used tool, it is becoming increasingly important to teach ultrasound techniques in medical school and residency and to graduate physicians competent in ultrasound technique and interpretation. There is significant literature from medical educators evaluating the feasibility of teaching ultrasound to medical students [4-9]. Ultrasound education for students in its first iterations consisted of demonstrating the technique in a large audience setting. Now, medical schools across the country have implemented an ultrasound curriculum that is integrated for students to learn scanning alongside the lecture material and to supplement anatomy and physiology lectures [10-14].

Dreher et al. evaluated the utility of head and neck scanning sessions for first-year medical students. Students first watched a head and neck ultrasound lecture, then participated in a hands-on session focused on identifying the tracheal rings, thyroid gland, carotid arteries, and jugular veins in live models. They completed pre- and post-session surveys to assess their self-perceived ability to obtain and interpret ultrasound images and the utility of ultrasound in the anatomy curriculum. 144 students completed both surveys, and there was a significant increase in student interest and self-perceived experience, comfort, and confidence in ultrasound skills (p < 0.001) as a result of this early introduction to ultrasonography [15]. Another study demonstrated a significant increase in student confidence levels in both ultrasound-naïve and ultrasound-experienced medical students after multimodal education with a didactic, live model scanning, and fine-needle aspiration (FNA) on gel phantoms [16]. Harvard Medical School also incorporated ultrasound into their first- and second-year curricula. First-year students used ultrasound to identify structures of the neck (i.e., thyroid, carotid artery, jugular vein) in their anatomy lab sessions. On the other hand, second-year students advanced to evaluating normal versus abnormal neck structures during their physical exam course, which also incorporated physical exam skills, such as palpating borders of the thyroid [17].

Other programs, such as the University of South Carolina School of Medicine, have developed a four-year integrated ultrasound curriculum. This includes head and neck ultrasound in the first and third year of medical school, with progress tracked by objective structured clinical examinations (OSCE). First-year students used B-mode to assess the thyroid, focusing on organ echotexture, nodules, cysts, measuring and labelling structures, and estimating thyroid lobe volume. Third-year students were presented in an OSCE with a “lump in the neck” and expected to obtain a history and physical exam as well as scan and measure the mass with ultrasound [18]. Furthermore, two studies also report a curricula for fourth-year students to provide hands-on training as they prepare for residency [6,9].

In recent years, Feilchenfeld et al. have critically examined the discourses that have led to the integration of ultrasound in medical education [19,20]. In one systematic review, this group used Foucauldian critical discourse analysis to compare the dominant rationales for integrating ultrasound in medical education with the empirical evidence in support of these rationales. They found that the current data does not support the integration of ultrasound in medical education, and the repetition of these rationales in literature is what substantiates its implementation [19]. This is an important criticism and is one of the impetuses for our study.

The thyroid ultrasound scanning sessions described in our study were incorporated into the endocrinology block of the first-year medical school curriculum during the 2018-2019 academic year to enhance the education of the anatomy, physiology, and pathology of the thyroid gland. Prior to this intervention, thyroid anatomy and pathophysiology education consisted of traditional didactic lectures and cadaver dissection labs. This study aims to evaluate the efficacy of this educational tool in the medical school curriculum by assessing student knowledge pre and post-session. The thyroid ultrasound scanning sessions were developed and taught by ultrasound proctors with supervision from curriculum block leaders and faculty from the Ultrasound Division of the Department of Emergency Medicine and the Endocrinology Division of the Department of Internal Medicine. Literature revealed our study to be novel in that while many studies evaluated student confidence levels pre- and post-scanning sessions, no study quantified and reported knowledge acquisition by thyroid-specific clinical questions that incorporated both clinical reasoning and ultrasound interpretation. We hypothesized that an integrated thyroid ultrasound scanning session could enhance medical student understanding of thyroid anatomy and pathophysiology.

## Materials and methods

Population

The study population included all first-year medical students enrolled in Part 1 of the Lead, Serve, Inspire (LSI) curriculum at the Ohio State University College of Medicine (OSUCOM) during the 2018-2019 academic year. While students were required to attend the learning session, they could choose to opt-out of participation in the research of this learning experience. The students were provided with an informed consent prior to their participation.

Study design

This was a prospective, single-center cohort (pre-experimental) study to evaluate student performance and knowledge acquisition using a pretest-posttest design. We obtained IRB approval to conduct this study (Study ID 2019E0254, approved by the Ohio State University). Each thyroid ultrasound session incorporated online learning in the way of narrated slideshows, didactic in-class instruction, and hands-on experience to reinforce the learning objectives. Additionally, an anonymous pretest and posttest using the electronic survey platform, SurveyMonkey, was used to assess the medical students’ learning from the ultrasound experience (Figures 5-10 in Appendix).

There was a total of eight 1-hour sessions to accommodate 26 to 27 medical students per session, accounting for 100% of the first-year student body. Students signed up for the session that best fits their schedule until the sessions were full. The sessions occurred on March 28th, March 29th, April 1st, and April 2nd of 2019 with two sessions each day.

Study procedure

Students received an email from the OSUCOM with information about the sessions and the voluntary nature of their participation in this study. Students were provided with a link to the pretest (Figures 5-7 in Appendix) five days prior to the ultrasound session to be completed before their corresponding session. By the time the students received the pretest link, they had already covered neck anatomy, thyroid pathophysiology, histology, and thyroid nodule workup through in-class lectures and online modules. They also dissected the neck in the cadaveric laboratory prior to the pretest as part of the Endocrinology block curriculum. The content of the pretest was covered in these learning modules.

The first question of the pretest was the informed consent, for which they had the option to choose between a “yes” or “no” response. Declining to participate in the study did not disqualify them from participating in the educational sessions. Answers to the pretest were linked to the answers gathered during the posttest. Once the links were established, the student responses were deidentified, thus rendering their answers anonymous. The posttest link was sent the same day of the student session. They had five days to access it after the session.

Each session was one hour long and had three stations: normal thyroid, thyroid with pathology, and FNA. Each station was staffed by a proctor, who was either a fourth-year medical student with neck ultrasound experience or an Emergency Medicine resident physician. All proctors were supervised by curriculum block leaders and clinical faculty from the Ultrasound Division of the Department of Emergency Medicine and the Endocrinology Division of the Department of Internal Medicine. All supervising physicians were credentialled with the Endocrine Certification in Neck Ultrasound for training in both diagnostic ultrasound and ultrasound-guided FNA. The role of proctors was to show the students how to do neck ultrasound and find anatomical landmarks with the probe before allowing them to scan the patients themselves. The supervising faculty expanded on the knowledge of the proctors by pointing out findings in the patient scans and discussing thyroid pathophysiology. The stations for normal thyroid and thyroid with pathology had three patient volunteers each. These patients were not compensated. There were one proctor and one faculty supervisor present per patient, and four to six students at each patient table at a time.

**Figure 1:**
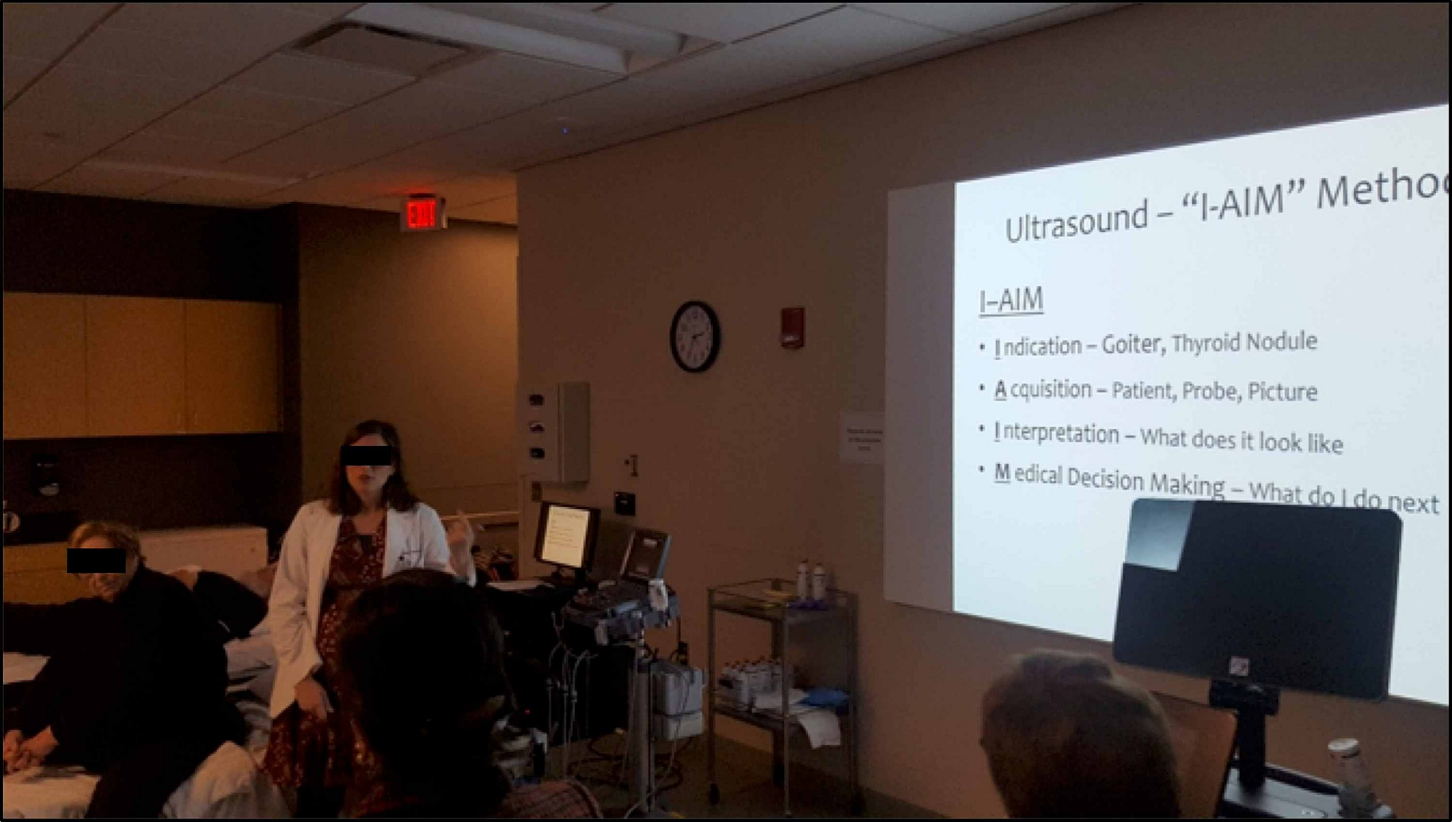
Introductory presentation covering objectives of the session, a basic introduction to ultrasound including the I-AIM technique, basic ultrasound physics, ultrasound probe types and motions, image orientation, and image description. I-IAM, indication, acquisition, interpretation, and medical decision making.

At the beginning of each session, all students received a five-minute presentation covering the objectives of the session, a basic introduction to ultrasound including the I-AIM (indication, acquisition, interpretation, and medical decision making) technique, ultrasound probe types and motions, image orientation, and image description (Figure 1) [21]. The students were familiar with basic ultrasound physics and ultrasound motions from prior ultrasound sessions offered in the curriculum. The second part of the presentation provided a brief overview of thyroid imaging and thyroid nodule workup that served as a review of the material already covered in in-class lectures during the Endocrinology block. This presentation was given by one of the authors of the study (Dr. Anisley Valenciaga), a fourth-year medical student with over three years of longitudinal ultrasound experience throughout medical school, including extensive extracurricular experience.

**Figure 2:**
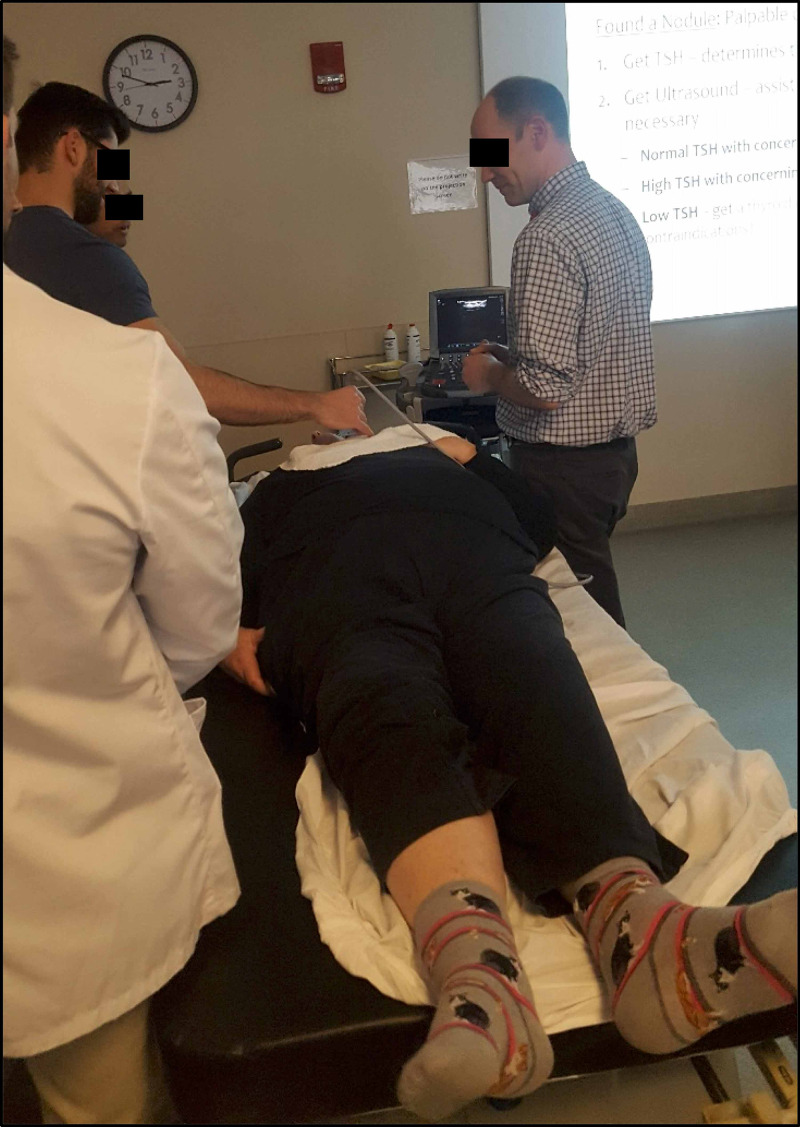
Student scanning volunteer patient with supervision from an endocrinologist.

After this presentation, the students were divided into two groups. Group 1 began the scanning session at the normal thyroid station, while Group 2 began at the station with thyroid pathology. Each station had three volunteer patients without or with thyroid pathology, respectively. Each patient table was staffed with one proctor and one supervising physician (Figure 2). Students had 20 minutes to scan the three subjects at the station they started with. There were four to six students at each patient table at any given time. After 20 minutes, the two groups switched stations and scanned for another 20 minutes at the new station, with three other subjects. The proctors showed the students how to use the ultrasound machine, taught them about ultrasound probe technique and patient position, and pointed out anatomical landmarks of the neck including trachea, sternocleidomastoid muscle, internal jugular vein, carotid artery, thyroid gland, and any nodules, if present. They were also shown how to use color Doppler to identify flow in the superior thyroid artery and/or in the nodule. Students then had the opportunity to practice these techniques. Clinical questions were also addressed at each station about the thyroid anatomy and pathophysiology by the faculty supervisors. Each student had the opportunity to scan all patients at both stations in the 20 minutes allotted for each station. More time was needed for the first patient, since the students were taken through proper scanning technique. Subsequent patient scans took less time for each student to accurately complete.

**Figure 3:**
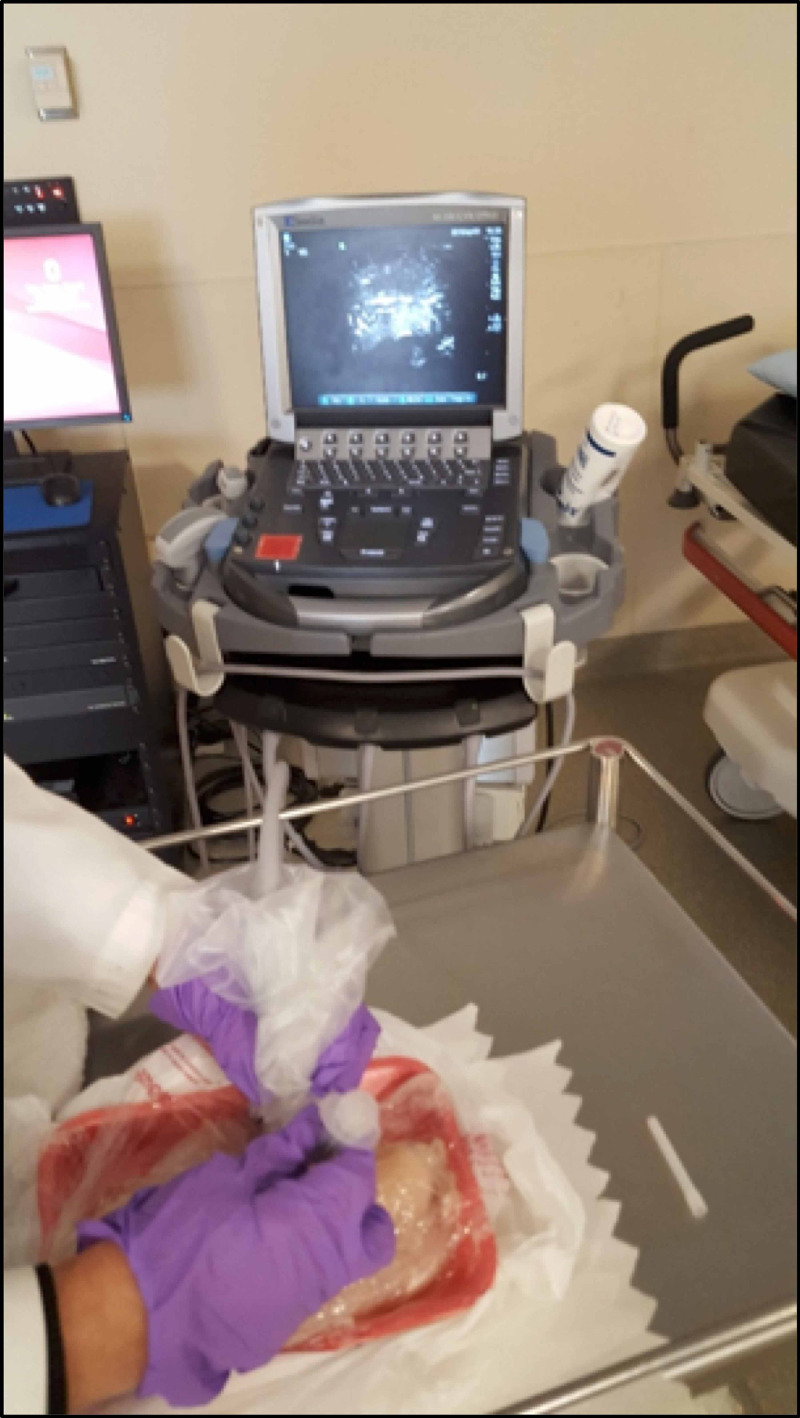
Ultrasound-guided fine-needle aspiration (FNA) being performed on a thyroid phantom.

After all students completed both the normal thyroid and pathologic thyroid stations, students gathered at the third station for 10 minutes where a thyroid disease case was discussed, including workup for a new thyroid nodule. An ultrasound-guided FNA was demonstrated in a thyroid phantom model (Figure 3). The last five minutes of the session included a debrief reviewing other sample cases. The posttest was distributed through the electronic survey tool one to two hours after the student’s thyroid ultrasound education session (Figures 8-10 in Appendix).

Equipment

In the Ohio State University's Clinical Skills Education and Assessment Center, SonoSite M-Turbo (SonoSite, Inc, Bothell, WA) ultrasound machines were used with L25x linear ultrasound transducers (SonoSite, Inc, Bothell, WA) with 13-6 MHz bandwidths.

Jamshidi® Menghini Soft Tissue Biopsy Needle/Syringe kits were used with probe covers on the transducers to biopsy the thyroid nodule ultrasound phantoms. A published novel low-cost thyroid ultrasound phantom was used for FNA demonstration [22]. It was assembled by using a chicken breast with pimento olives embedded inside to simulate nodules. Disposable gloves were worn. A scalpel was used to create three 1 cm incisions on the inferior surface of the chicken breast, and the incisions were bluntly dissected with a gloved finger. One pimento olive was placed in each incision to simulate an intermediately suspicious mass sonographically. The return of the red pimento on FNA indicates a successful sampling. The loaded chicken breast was then placed inside a kitchen zipper plastic bag, the air was squeezed out, and the bag was tightly zipped closed.

Measurement and data analysis

For each session, we assessed knowledge before and after the sessions using a content-related pretest and posttest covering anatomy and clinical scenarios. The knowledge tests provide students with a sense of how well they learned from the sessions.

The students’ pretest and posttest scores were gathered and matched by a data analyst who had no supervisory authority over the medical students. At the point in time that pretests and posttests were matched, the identifying information (email addresses) was removed. Scoring of the tests was conducted using IBM SPSS® Statistics software. A dependent (paired) t-test comparing pretest and posttest along with analysis of associated effect size (Cohen’s D) was calculated using IBM SPSS® Version 25 (IBM Corp., Armonk, NY). Item analyses were performed on the tests to evaluate the test items for quality and appropriate levels of difficulty.

## Results

All 209 first-year students attended the scanning sessions. They were able to scan healthy subjects as well as subjects with thyroid pathology. The sessions were interactive, with students asking questions and actively participating (Figures 1-3).

**Figure 4:**
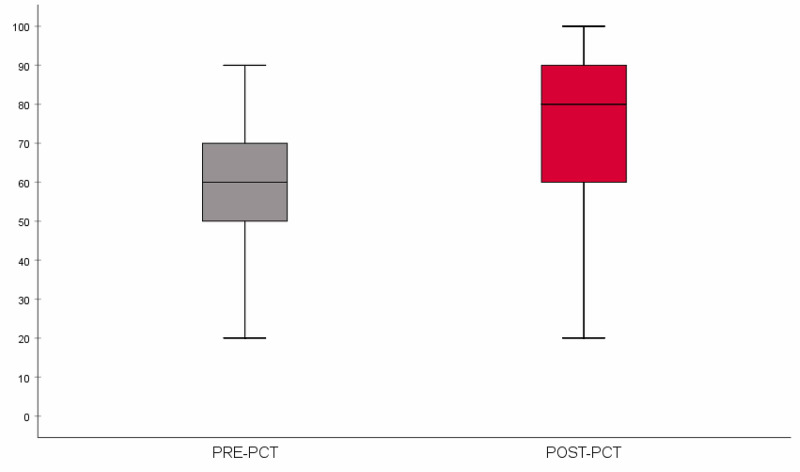
Boxplot comparing the distribution of pretest and posttest scores (pretest n = 114; posttest n = 114). The horizontal line within the box represents the median score.

**Figure 5:**
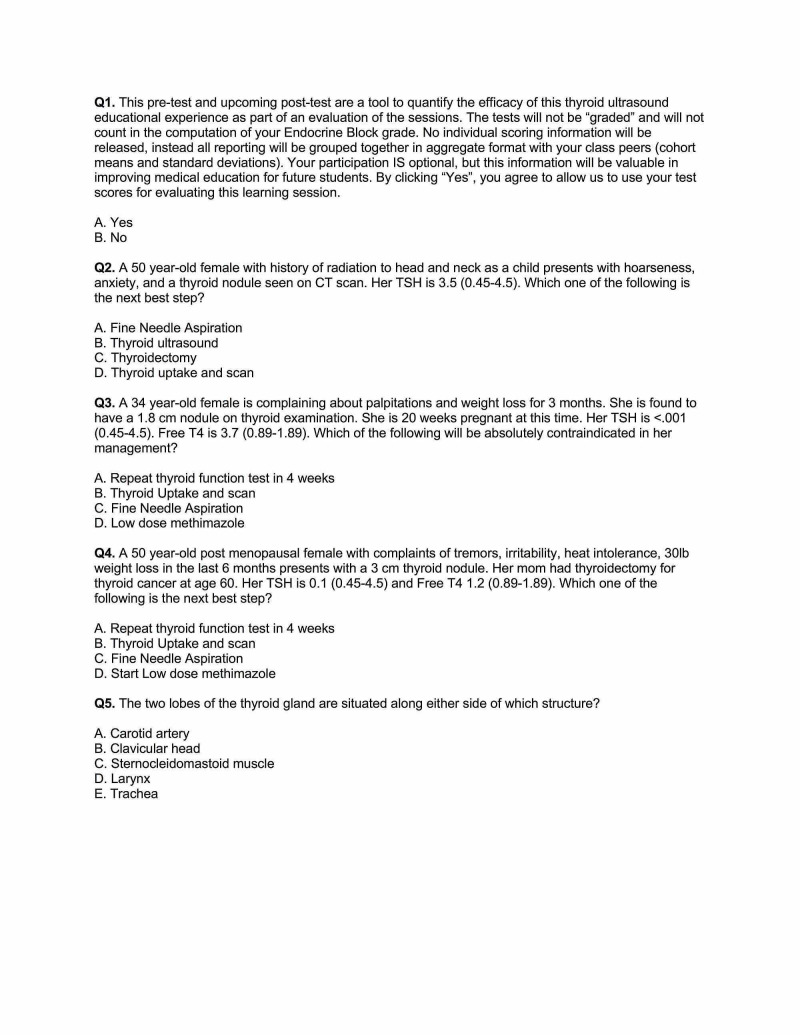
Pretest questions (1/3). The first question asks consent for participation in this study.

**Figure 6:**
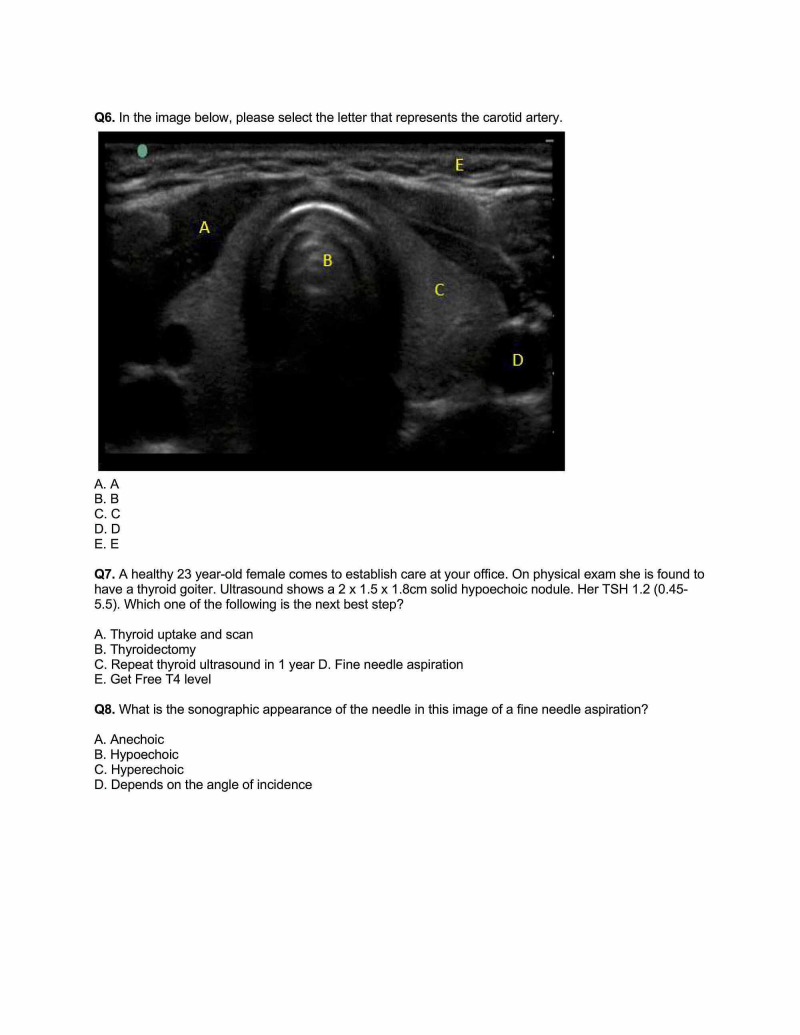
Pretest questions (2/3).

**Figure 7:**
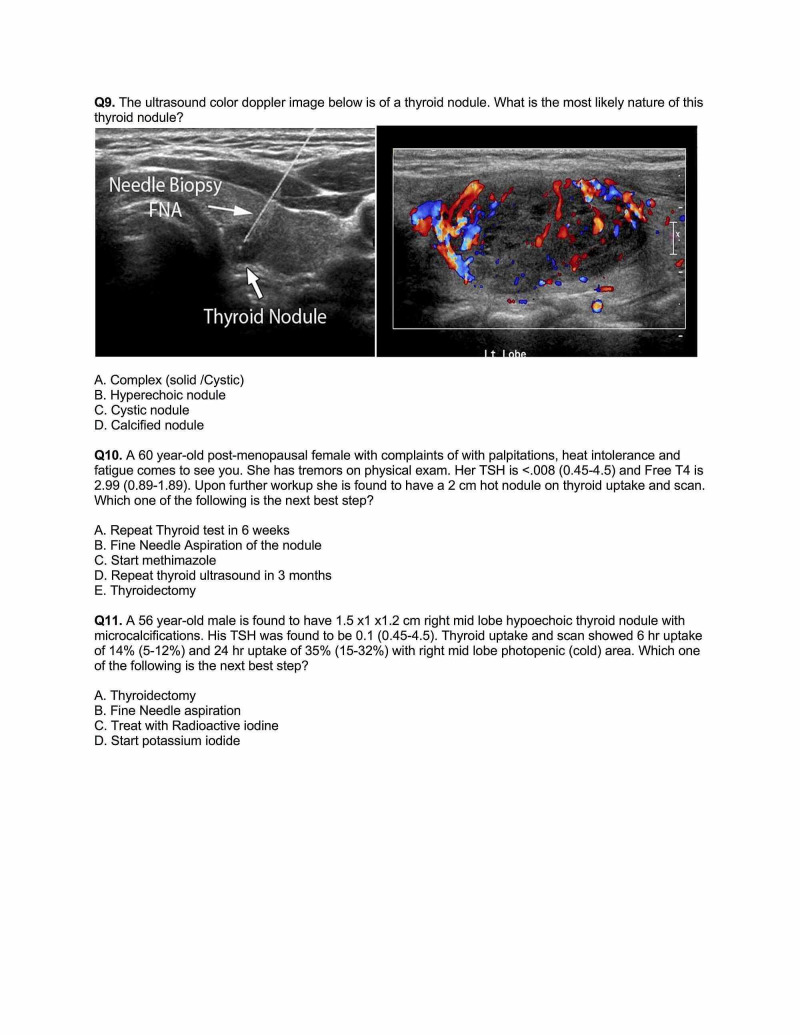
Pretest questions (3/3).

**Figure 8:**
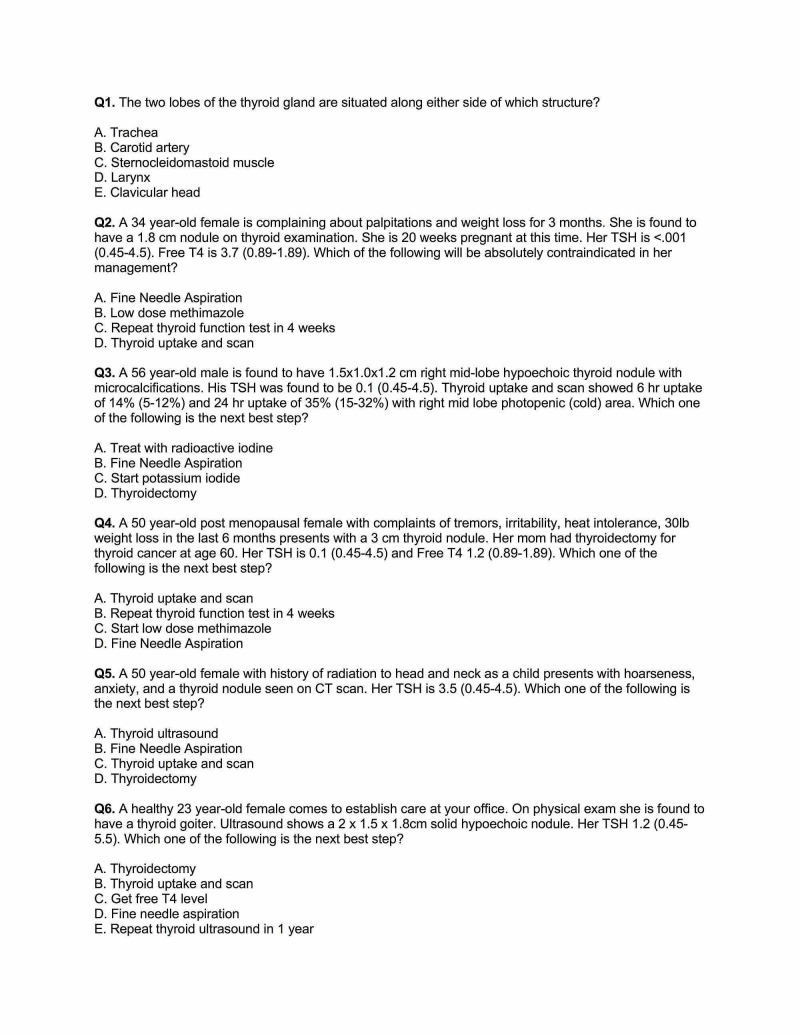
Posttest questions (1/3).

**Figure 9:**
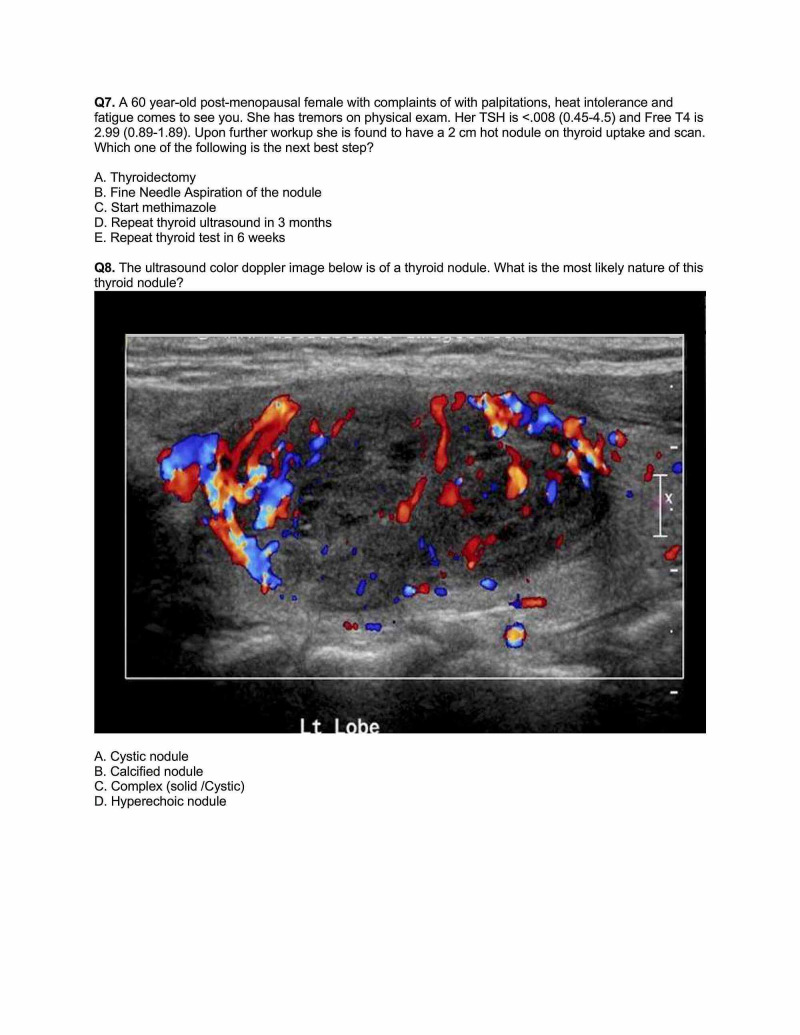
Posttest questions (2/3).

**Figure 10:**
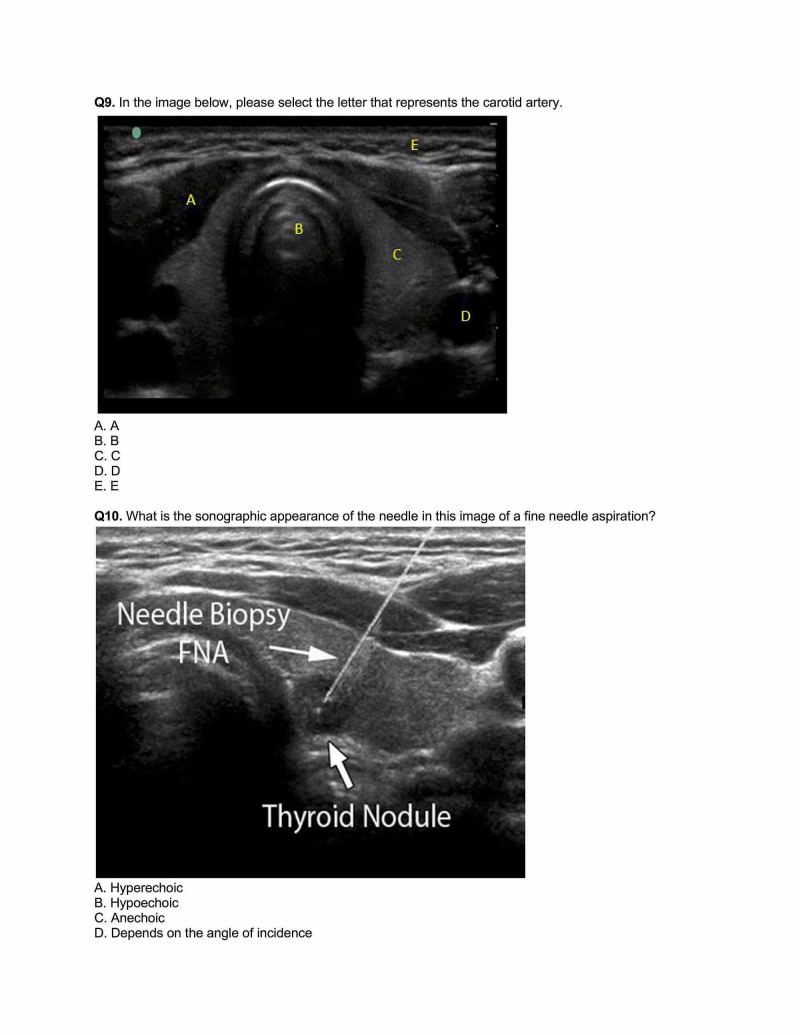
Posttest questions (3/3).

Out of the 209 students who participated in the ultrasound sessions, 144 (68.9%) consented to participate in the research project (Table 1). Of these students, 114 (79.2%) completed both the pretest and posttest (Table 2). Test data from the 114 students showed a mean score of 57.5% ± 14.6% in the pretest and 73.9% ± 17.4% in the posttest, with a difference of 16.5% ± 19.6% (p < 0.001) between both tests (Table 3, Figure 4).

**Table 1:** Student consent to the research project.

Consent	Frequency	Percent
YES	144	68.9
NO	65	31.1
Total	209	100.0

**Table 2:** Consent by participation.

	CONSENT		Total
	YES	NO	
Pre Only	27	1	28
Post Only	1	18	19
Both Pre and Post	114	18	132
Neither Pre nor Post	2	28	30
Total	144	65	209

**Table 3:** Test results for respondents who took both tests and consented. *Cohen’s D effect size is interpreted as: < 0.1 = no effect; 0.2-0.4 = small effect; 0.5-0.7 = medium effect; > 0.8 = large effect.

	Mean	Standard Deviation
Pretest	57.5	14.6
Posttest	73.9	17.4
Difference	+16.5	19.6
t-test	t = 9.001; df = 113; p < 0.001; es=1.027*	

 

## Discussion

A multimodal, interactive thyroid ultrasound scanning session integrated into the first-year medical school curriculum is an effective tool to improve students’ knowledge of thyroid anatomy, pathophysiology, and diagnostic workup of thyroid nodules. Its efficacy is evidenced by the significant improvement in posttest versus pretest scores of participating students. This study is unique in that we quantify knowledge acquisition through thyroid-specific clinical questions that incorporate both clinical reasoning and ultrasound interpretation.

The in vivo nature of this scanning session offered a more controlled environment for education, scanning competency, and user confidence than a real-life clinical setting. Patients volunteered their time for medical student education, so they were amenable to repeated scans, repositioning, and the inexperienced technician. Also integral to the success of this session, the proctors and faculty supervisors demonstrated ultrasound technique and thyroid nodule measurement, as well as integrated the didactic session, ultrasound interpretation, and clinical scenarios. Therefore, our session provided an optimal learning environment for our medical students. Gaining comfort in measuring, describing, and diagnosing lesions prior to seeing patients in clinic is advantageous to both the patient and provider [22]. Future studies may explore other ways to quantify the impact of this session. We could explore sustained knowledge gain by adding a test the following academic year for the same students who participated. We can also include hands-on assessments in the future to evaluate the knowledge gain as well. If used for resident training, we could attempt to quantify the impact of this intervention by determining the number of actual in-office scanning sessions one of our sessions represents. We could then define our session in terms years of residency training it would take to acquire an equal amount of competency [23].

In addition, future projects should consider surveying students for their opinions after the session. Verbal feedback after the sessions indicated that the students enjoyed the hands-on experience and the interactive case discussion; however, a formal survey would be beneficial to elicit more opinions, quantify student confidence, and gather insight into why some students who consent to participate do not take the pretest and posttest. Furthermore, due to the brief nature of the sessions, the students were not able to practice an FNA biopsy with the thyroid phantom. We may extend the session length in the future to accommodate this. Using phantom models for FNAs allows for repetition and improvement in technique without risk of harm to patients. The nature of the phantom used also encourages safe practices technique, similar to in vivo human tissue, because of the biohazard concern of the uncooked chicken. Later studies may also include assessing the accuracy of medical students in identifying and measuring pathology in real patients and performing FNAs on thyroid phantoms.

We would be remiss to not discuss the counterpoints presented by Feilchenfeld et al. regarding the rapid growth of ultrasound in the medical school curricula [19,20]. These papers challenge the rationales presented by proponents of ultrasound in medical education. Namely, they state that there is insufficient evidence to make the claim that ultrasound leads to a better understanding of anatomy [19]. Likewise, one randomized controlled trial of second-year medical students at a single institution compared the effects of cadaver-only versus cadaver plus ultrasound versus cadaver plus arthroscopy on shoulder and knee anatomy education. They found no difference in anatomical knowledge between the ultrasound and cadaver-only groups, and arthroscopy was superior to ultrasound in augmenting education of shoulder anatomy [24]. Contrarily, our findings support the claim that this thyroid ultrasound session not only increases the knowledge of thyroid anatomy, but also knowledge of the pathophysiology of the thyroid gland. As ultrasound is standard of care for diagnosing and working up thyroid nodules, we contend that it is more important for the anatomy of this structure to be taught with an ultrasound component. Furthermore, the conflicting evidence presented by this systematic review begs additional research on the efficacy of integrating ultrasound into medical school curricula, which this study does [19]. We present positive results in support of the implementation of a thyroid ultrasound session to augment thyroid anatomy education. Despite this, one future direction of our study is to conduct a randomized controlled trial, specifically, to split the students into two groups, cadaver-only and cadaver plus ultrasound session, in order to gain a better understanding of the knowledge gained by our session.

Limitations

Limitations of the study include the absence of a control group of students not participating in the scanning sessions but taking the pretest and posttest. Because these ultrasound sessions were a required part of the curriculum, no students were absent from them, making it challenging to obtain a control group. In the future, we may try to strengthen our study by dividing the students into two groups, cadaver-only and cadaver plus ultrasound sessions, to gain a better understanding of the knowledge gained by our session. Another limitation was the lack of qualitative feedback from students. We receive verbal feedback from the participants, which was valuable for future iterations. As mentioned above, the students were given in-class lectures and online modules that covered knowledge on thyroid anatomy, histology, and pathophysiology; however, we do not know whether all students prepared before the sessions by participating in class and studying the online modules. Therefore, we do not know if the students who did best on the pretest had more preparation. We can include an anonymous survey in future studies that asks the participants to acknowledge what they did to prepare before the pretest. That being said, it can be stated that the pretest functioned as a readiness assessment test of sort, as it tested material previously covered in curriculum lectures and the cadaver lab. Furthermore, these sessions occurred in March and April 2019. The second iteration was planned to occur in April 2020 but was cancelled due to the COVID-19 pandemic, limiting us to a single year of data.

We also acknowledge that a limitation in translating this study lies in the fact that some institutions might find it difficult to secure patients with thyroid pathology to volunteer for the sessions. We suggest that these institutions carry out the sessions with normal thyroid subjects to allow the students to at least see normal neck anatomy on ultrasound. They can then go over thyroid pathology on ultrasound in lecture form or other learning styles that suit the institution. In cases of institutions with limited proctors and faculty trained to do neck ultrasound and able to teach at the stations, a suggestion might be to spread the scanning sessions throughout the year as to have fewer students per session. Future directions include using other ways to assess hands-on ultrasound skills, such as finding, describing, and measuring nodules, and conducting a follow-up test several months later to demonstrate long-term gains in knowledge.

## Conclusions

Bedside ultrasound is an increasingly common point-of-care measure used by physicians and trainees to aid in the diagnosis and treatment of multiple diseases at bedside. As such, it is integral to teach ultrasound techniques in medical school and to graduate physicians competent in ultrasound technique and interpretation. Overall, our multimodal thyroid ultrasound scanning session was a successful learning experience for first-year medical students that offered a quantified thyroid-specific growth of knowledge. This session should continue to be offered in future years and potentially be adopted by other medical colleges to enhance their endocrine curricula.
